# Mucins as a New Frontier in Pulmonary Fibrosis

**DOI:** 10.3390/jcm8091447

**Published:** 2019-09-11

**Authors:** Beatriz Ballester, Javier Milara, Julio Cortijo

**Affiliations:** 1 Department of Pharmacology, Faculty of Medicine, University of Valencia, 46010 Valencia, Spain; 2 CIBERES, Health Institute Carlos III, 46010 Valencia, Spain; 3 Institute of Health Research-INCLIVA, 46010 Valencia, Spain; 4 Research and teaching Unit, University General Hospital Consortium of Valencia, 46014 Valencia, Spain

**Keywords:** idiopathic pulmonary fibrosis (IPF), biomarkers, mucins

## Abstract

Idiopathic pulmonary fibrosis (IPF) is the most common idiopathic interstitial pulmonary disease with a median survival of 3–5 years after diagnosis. Recent evidence identifies mucins as key effectors in cell growth and tissue remodeling processes compatible with the processes observed in IPF. Mucins are classified in two groups depending on whether they are secreted (secreted mucins) or tethered to cell membranes (transmembrane mucins). Secreted mucins (MUC2, MUC5AC, MUC5B, MUC6-8 and MUC19) are released to the extracellular medium and recent evidence has shown that a promoter polymorphism in the secreted mucin MUC5B is associated with IPF risk. Otherwise, transmembrane mucins (MUC1, MUC3, MUC4, MUC12-17 and MUC20) have a receptor-like structure, sensing the external environment and activating intracellular signal transduction pathways essential for mucosal maintenance and damage repair. In this context, the extracellular domain can be released to the external environment by metalloproteinase action, increased in IPF, thus activating fibrotic processes. For example, several studies have reported increased serum extracellular secreted KL6/MUC1 during IPF acute exacerbation. Moreover, MUC1 and MUC4 overexpression in the main IPF cells has been observed. In this review we summarize the current knowledge of mucins as promising druggable targets for IPF.

## 1. Introduction

Interstitial lung diseases (ILDs) are a group of disorders characterized by cellular proliferation, interstitial inflammation, fibrosis, or a combination of these conditions within the alveolar wall [1]. If no cause is identified, patients receive a diagnosis of idiopathic interstitial pneumonia (IIP), with interstitial fibrosis being the predominant phenotype [1]. The most common type of IIP is idiopathic pulmonary fibrosis (IPF) [1], which is defined as a type of chronic, progressive fibrosing interstitial pneumonia of unknown cause, occurring primarily in older adults, limited to the lungs and associated with the histopathological and/or radiological pattern of usual interstitial pneumonia [2].

The course of IPF is variable and somewhat unpredictable. Despite the recent approval of two treatments (pirfenidone and nintedanib), the median survival time from diagnosis is 3–5 years [3]. Therefore, biomarkers of IPF are essential to predict disease progression and treatment response, as well as for differential diagnosis [4]. IPF biomarkers can be categorized into three groups, namely biomarkers associated with alveolar epithelial cell dysfunction (such as the Krebs von den Lungen-6 (KL-6) antigen, carbohydrate antigen (CA)15-3, CA125, the mucin MUC5B and surfactant proteins A and D), biomarkers associated with extracellular matrix remodelling and fibroproliferation (such as matrix metalloproteinase-1 (MMP-1) and -7 (MMP-7), lysyl oxidase-like 2 and periostin), and biomarkers related to immune dysfunction (such as CC chemokine ligand-18 and YKL-40) [5,6,7,8,9] (Table 1).

KL-6 is a high-molecular-weight glycoprotein classified as a human transmembrane MUC1. Several studies have reported increased serum KL-6 levels during acute IPF exacerbation and a recent study demonstrated that serial increases in serum KL-6 levels are associated with a rapid decline in predicted forced vital capacity (FVC), and further demonstrated that higher KL-6 levels are correlated with lower survival rates [11]. The use of MUC5B as an IPF biomarker is based on a common gain-of-function promoter variant (*rs35705950*) associated with IPF development. This polymorphism has been reported in 30%–35% of IPF patients [20]. These findings indicate the importance of lung mucins in the development of IPF disease, suggesting that lung mucins may offer an effective alternative therapeutic target for this disease. This review summarizes the current state of lung mucin research related to pulmonary fibrosis and provides an assessment of the potential of mucins as treatment targets in this disease.

## 2. Overview of Lung Mucins

Airway mucus is the layer that covers, protects and lubricates the respiratory tract. This mucus is composed of water, ions, lung secretions, serum protein transudates, anti-microbial proteins and mucus glycoproteins (mucins) [35,36,37]. Mucins are high-molecular-weight glycoproteins produced by surface epithelium goblet cells, submucosal glands, and serous cells. They are characterized by an extensively glycosylated protein structure with a variable number of amino acid tandem repeats (TRs), which are especially rich in serine, threonine and proline (potential sites for O-glycosylation) [38].

Mucins are classified into two groups depending whether they are secreted (secreted mucins) or tethered to epithelial cell membranes (transmembrane mucins (TM)). Secreted mucins are further subdivided into gel-forming and non-gel-forming mucins [39].

Mucins are encoded by MUC genes. To date, 21 human MUC genes have been identified (Table 2), of which 16 have been identified in the lung: 10 TM mucins (*MUC1, MUC4, MUC12, MUC13, MUC14, MUC15, MUC16, MUC20, MUC21, MUC22*); 4 secreted gel-forming mucins (*MUC2, MUC5AC, MUC5B, MUC19*); and 2 secreted, non-gel-forming mucins (*MUC7, MUC8*)) [37,40,41]. Together, MUC5AC and MUC5B account for approximately 90% of the mucin content of sputum. Nearly all of the remaining 10% is made up of three membrane-tethered mucins: MUC1, MUC4, and MUC16 [42].

### 2.1. Secreted Mucins

**Figure 1:**
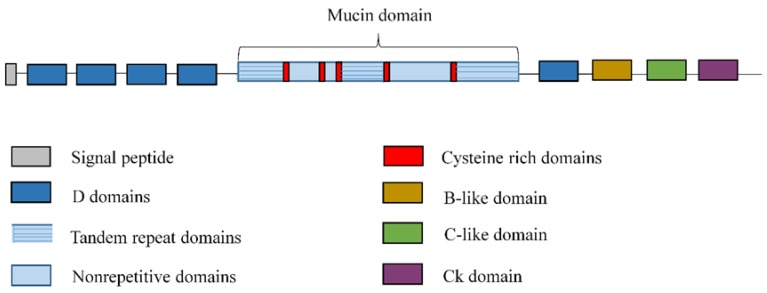
General structure of secreted mucins. Secreted mucins are composed of a large central exon, containing the entire tandem repeat domain, nonrepetitive domains and at least five important cysteine-rich domains, and flanking 5′ and 3′ regions vonWillebrand factor (vWF)-like domains (D domains, the B domain, the C domain, and the CK domain).

Secreted mucins are the most abundant glycoprotein component of mucus [38]. They are secreted into the extracellular space and share a similar gene structure, comprised of a large central exon containing the entire TR domain, nonrepetitive domains and at least five important cysteine-rich domains that play a role in disulfide-mediated polymer formation, and the flanking 5′ and 3′ regions [19,43] (Figure 1). The sequence and length of the TR units are unique to each mucin and may include variable number tandem repeat (VNTR)-type polymorphisms. The major function of these TR domains is to provide a scaffold for O-linked carbohydrates [19].

Secreted gel-forming mucins are characterized by their high molecular weight (5–40 MDa), large size (600–900 nm), high proportion of glycosylation (50%–80%) and the capacity to oligomerise and build up viscoelastic gel, which covers the respiratory epithelium by forming a matrix where bacteria are trapped, thus providing a barrier function [19,37,41]. They also capture, retain and release biologically active molecules such as cytokines, growth factors and trefoil factors. These association and dissociation properties may allow mucins to regulate inflammation and immune responses, and to influence post-injury epithelial repair [37]. Moreover, gel-forming mucins impart a lubricant property to the mucus [41]. By contrast, secreted non-gel-forming mucins cannot oligomerise and are observed as monomers [41].

As noted above, MUC5AC and MUC5B are the major, and best-described, secreted gel-forming mucins. Elevated levels of MUC5AC and MUC5B have been reported in asthma patients [44], and elevated MUC5B levels have been observed in patients with chronic obstructive pulmonary disease (COPD) [45]. However, the functions of MUC2 and MUC19 in the lung are not known, and nor are those of MUC7 and MUC8. Nevertheless, decreased expression of MUC2 has been observed in patients with COPD [45], and although it has not been investigated in airway mucus, the secreted non-gel-forming mucin MUC7 has been well characterized in saliva. The N terminus of MUC7 reportedly contains a histatin-like domain that confers antifungal activity and interacts with bacteria. Thus, MUC7 is clearly an important innate defence protein [19]. Various authors have considered innate immune mechanisms to contribute to lung fibrogenesis [46,47], and genetic variants of the regulator of innate immune responses toll-interacting protein (TOLLIP) have been associated with sporadic IPF [48]. These findings suggest an essential role of MUC7 in IPF disease. Furthermore, it has been reported that MUC7*5 allelic polymorphism, which has nine fewer potential O-glycosylation sites, is significantly associated with decreased risk of asthma in African-Americans [49] and Northern Europeans [50]. These results suggest that this association is related to allelic differences in bacterial interaction, as the glycosylated domain in the N terminus is thought to be responsible, at least in part, for bacterial binding [51].

### 2.2. Transmembrane Mucins

**Figure 2:**
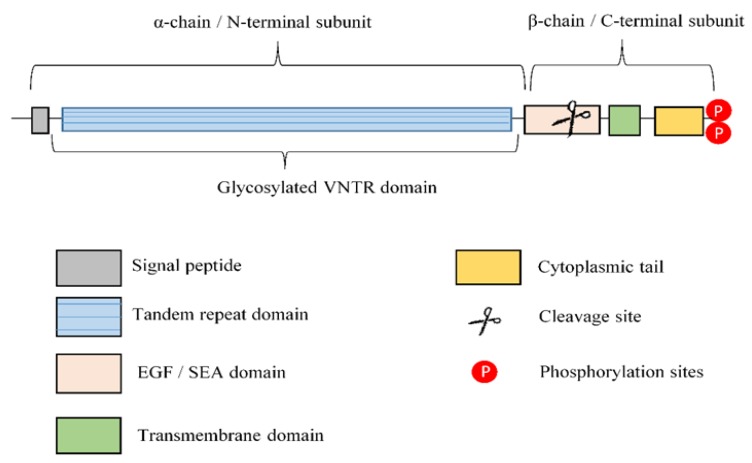
General structure of the transmembrane mucins. Transmembrane mucins are composed of two subunits: α chain or N-terminal subunit and β chain or C-terminal subunit, based on the putative proteolytic cleavage site in the epidermal growth factor/sperm protein, enterokinase and agrin (EGF/SEA) domain. The α-chain is extracellular and predominantly composed of variable number of tandem repeats (VNTR) highly glycosylated. The β-chain consists of a short extracellular region (containing either SEA domain or EGF-like domain), single transmembrane (TM) domain, and the cytoplasmic tail, which contains multiple phosphorylation sites.

TM mucins are large glycoproteins that localize to the apical surfaces of epithelial cells exposed to relatively harsh environments [35]. Cell surface mucins are typically composed of dimers of two dissimilar subunits (α and β chains), held together by non-covalent sodium dodecyl sulfate-labile bonds [42]. The larger subunit (α-chain) is wholly extracellular and heavily glycosylated. In contrast, the smaller subunit (β-chain) consists of a short extracellular region (containing either sperm protein, enterokinase and agrin (SEA) domain or epidermal growth factor (EGF)-like domain), the single-pass TM domain, and the cytoplasmic tail (CT) [52] (Figure 2). Similar to classical innate immune receptors, α-chain senses the external environment and activates intracellular signal transduction pathways mediated by the CT, making it essential for mucosal maintenance and damage repair [35]. It has been hypothesized that extracellular mucin domains are released in response to mechanical force; interactions with microbes; alterations in pH, ionic concentration, or hydration [53]; and inflammatory stimuli such as tumour necrosis factor-α (TNF-α) and neutrophil elastase [54]. The high level of glycosylation in this extracellular domain shields the protein backbone of the extracellular domain from proteolytic attack by bacteria and host proteases, and contributes to barrier formation. Furthermore, the intracellular tails of all TM mucins contain putative phosphorylation sites, but these tails are dissimilar in sequence and length and do not contain any conserved domains. Together, these observations suggest a high degree of functional divergence and, most likely, signalling specificity among TM mucins [35]. Nevertheless, recent evidence in cancer studies has indicated that TM mucins are key effectors in the cell growth, proliferation, apoptosis, and epithelial to mesenchymal transition (EMT) processes [52,55], which is consistent with the processes observed in IPF. As noted above, MUC1, MUC4 and MUC16 are the predominant TM mucins in the lung.

## 3. Mucins as Potential Idiopathic Pulmonary Fibrosis (IPF) Drug Targets

Several lines of evidence link mucins to IPF disease, suggesting that they are key effectors of the disease. However, the exact role of mucins in IPF development processes remains unknown, as do their activation mechanisms and ability to mediate intracellular signalling. Thus, knowledge of these activation processes, and of intracellular fibrotic processes mediated by mucins, would be helpful for establishing IPF diagnostic tools, to elucidate IPF development and to identify promising IPF drug targets (Table 3).

### 3.1. Mucin 5B

MUC5B is mainly produced in mucous cells of the submucosal glands [19]. Thus, it may be involved in the response to chronic insults, playing a role in infection and inflammation [19].

**Figure 3:**
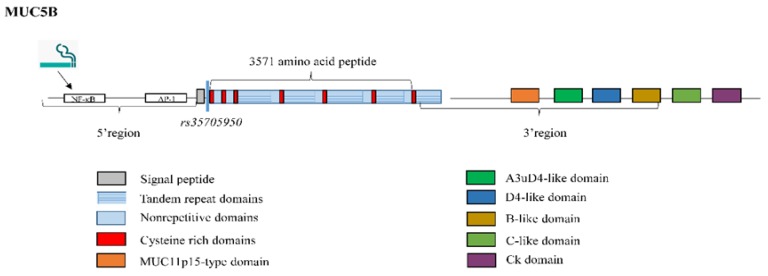
Structure of mucin MUC5B. The *MUC5B* gene has a large central exon which codes for a 3571-amino acid peptide and is constituted by tandem repeat, non-repetitive and cysteine rich subdomains. Most of these subdomains are similar to each other, thus forming four super repeat units consituded by a tandem repeat region, a unique sequence and a cysteine-rich region. 7 cysteine-rich regions are observed in total. The 3′ flanking region includes six subdomains (MUC11p15-type domain, A3uD4-like domain, D4-like domain, B-like domain, C-like domain, and CK domain). Nuclear factor kappa B (NF-κB) and activating protein 1 (AP-1) binding motifs have been revealed into the 5′-flanking region, inducing *MUC5B* expression. Cigarette smoking induces *MUC5B* promoter activation and gene expression at least in part by nuclear factor kappa B (NF-*κ*B). *rs35705950* is a common IPF polymorphism located 3-kb upstream of the *MUC5B* transcription start site.

The *MUC5B* gene has a particularly large central exon of 10,713 base pairs (bp), encoding a 3571-amino acid peptide [74]. This single large exon contains the entire TR domain; 19 subdomains have been identified [75], most of which are similar to each other, forming four super-repeat units of 528 amino acid residues that constitute a TR region, a unique sequence and a cysteine-rich region [41]. This central domain shows little variation in length among individuals [76] and acts as a scaffold for O-linked carbohydrates. MUC5B mucin has been reported to occur in two glycoforms, referred to as the low- and high-charge glycoforms [77]. Desseyn and associates [78] suggested that the remaining 3886 bp upstream of this large central exon comprised 29 exons, while the 3′ region of MUC5B was composed of 18 exons ranging in size from 32 to 781 bp [79], including six subdomains (MUC11p15-type domain, a 56-amino acid domain similar to the A3uD4 domain (located between the A3 and D4 domains in von Willebrand factor (vWF)), D4-like domain, B-like domain, C-like domain, and CK domain [79]) (Figure 3).

Several putative motifs for binding of transcriptional factors have been revealed in the DNA fragment containing the MUC5B 5′-flanking region. For example, the transcriptional factors nuclear factor kappa B (NF-κB) and activating protein 1 (AP-1) [75], which bind to the MUC5B promoter and subsequently induce MUC5B expression [80] (Figure 3). Interestingly, elevated levels of both transcription factors are involved in the expression of cytokines in T cells during lung injury and fibrosis, thus playing a pivotal role in IPF disease [81]. It has been reported that one potential IPF risk factor, cigarette smoking [2], induces MUC5B promoter activation and gene expression, and that this activation is mediated at least in part by NF-κB [82] (Figure 3).

MUC5B expression has been found to be 14.1 times higher in subjects with versus without IPF [20], and it is localized to IPF lesions in the distal airways, respiratory bronchioles, and honeycomb cysts [20,57]. The latter is a mucin-containing structure in the IPF lung [83] and MUC5B is the dominant gel-forming mucin accumulated in it, contributing to impair alveolar gas exchange [57]. In mice, it has been recently demonstrated that MUC5B overexpression in bronchoalveolar epithelia is related to impaired mucociliary clearance (MCC), indicating excessive retention of inhaled substances (air pollutants, particles and chemicals from cigarette smoke, microorganisms, etc.) or endogenous inflammatory debris, which causes a reactive or regenerative fibrotic response localized to the bronchoalveolar region and promotes lung fibrosis development [84,85]. In this context, MUC5B is required for controlling infections in the airways and middle ear, and for maintaining immune homeostasis in the lungs [86]. Excessive production of MUC5B presents a challenge to proper mucus hydration and cilia function [85]. Moreover, down-regulation of MUC5B has been reported to profoundly diminish proliferation, migration and invasion of human gastrointestinal cancer cells, where this reduction is mediated, at least in part, by alteration of the Wnt/β-catenin pathway and consequent reduction of β-catenin expression [87]; this is one of the key effector pathways in IPF disease [88].

The most important genetic risk factor for IPF is a common variant (*rs35705950*) located on the p-terminus of chromosome 11, 3 kb upstream of the MUC5B transcription start site. In fact, a Genome-wide association study (GWAS) found that 38% of subjects with IPF had a minor (T) allele of the single-nucleotide polymorphism (SNP) *rs35705950*. Moreover, this polymorphism was also associated with 34% subjects with familial interstitial pneumonia and it is linked to 37.4-fold MUC5B upregulation in the lung in unaffected subjects and 5.7-fold MUC5B upregulation in patients with IPF [20,89]. Interestingly, Chen et al., [90] recently demonstrated that IPF chronic endoplasmic reticulum stress differentially upregulates MUC5B expression in the context of the MUC5B promoter *rs35705950* variant through the ERN2-XBP1S pathway. Moreover, MUC5B overexpression in the bronchiolo-alveolar epithelia is enhanced by the MUC5B promoter variant *rs35705950* [91]. Finally, and surprisingly, IPF subjects with the MUC5B promoter variant *rs35705950* show improved prognosis, lower bacterial burden and improved survival compared to those without this variant [56,92]. Therefore, while the minor (T) allele is a risk factor for development of IPF, it also confers a survival advantage among patients with IPF [56]. This finding suggests that the MUC5B variant can be used either to identify individuals earlier in the course of the disease, or to differentiate pathophysiologic subtypes of IPF [93]. The use of this MUC5B promoter variant to identify individuals in the preclinical stages of pulmonary fibrosis may be ideal for early interventions focused on avoiding the development of extensive, permanent and irreversible lung remodelling.

### 3.2. Mucin 5AC

MUC5AC is produced mainly in the goblet cells of the surface epithelium [19]. Therefore, it may be an acute-response mucin produced as a result of insults to upper airway surfaces.

**Figure 4:**
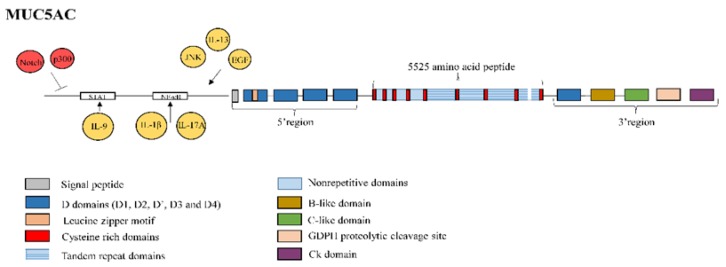
Structure of mucin MUC5AC. MUC5AC is a polymeric mucin made of 5,525 amino acids. The 5′-flanking region consists of the cysteine rich domains D1, D2, D’ and D3 and a putative leucine zipper motif. The central region is encoded by a single large exon containing 9 cysteine rich domains of which cysteine 1 to cysteine 5 domains are interspersed by nonrepetitive sequences and cysteine 5 to Cysteine 9 domains are interspersed by four tandem repeat domains. Only the last repetitive domain shows a slight variation in length. The 3′-flanking region consists of the cysteine rich domains D4, B, C and cysteine knot (CK). Moreover, a GDPH (Gly-Asp-Pro-His) autocatalytic proteolytic cleavage site is also present in this region. MUC5AC promoter activity can be downregulated by the histone acetyltransferase p300 and the Notch signalling pathway. MUC5AC promoter activity can be upregulated by autophagy, which is defective in IPF, through phosphorylation of Jun N-terminal kinase (JNK) and c-Jun, by interleukin (IL)-13, and by epidermal growth factor (EGF). IL-1β and IL-17A, through nuclear factor kappa B (NF-kB) binding site in the MUC5AC promoter, and IL-9, via the Janus kinase/signal transducers and activators of transcription (JAK/STAT) patway, also upregulate MUC5AC.

MUC5AC is a polymeric mucin comprising 5525 amino acids, which contains heavily glycosylated mucin-like domains rich in proline, threonine and serine (PTS) residues interrupted by poorly glycosylated cysteine-rich domains. The 5′ flanking region consists of the cysteine-rich domains D1, D2, D and D3, which show sequence similarity with the Von Willebrand factor (vWF), as well as a putative leucine zipper motif. The central region encodes a single large exon containing nine poorly glycosylated cysteine-rich domains, in which cysteines 1–5 are interspersed with heavily glycosylated mucin-like domains rich in PTS residues without any repetitive sequences. Cysteine domains 5–9 are interspersed with four TR domains of eight amino acid residues [41]. The central domain shows little length variation among individuals, with only one of the repetitive domains in MUC5AC showing a slight difference in length [76]. Finally, the 3′-flanking region consists of the cysteine-rich vWF-like domains D4, B, C, and the cysteine knot. In addition, a GDPH (Gly-Asp-Pro-His) autocatalytic proteolytic cleavage site is present in this region (Figure 4). The cysteine knot domain mediates dimerization through an autocatalytic process [41].

MUC5AC is the main mucin observed in healthy airway secretions [19], and the major airway mucin produced by goblet cells lining the airways [94]. It is involved in epithelial wound healing after mucosal injury [95] and activation of the cellular stress, damage and repair pathways, suggesting a key role in resolving the disrupted homeostasis that characterises IPF [96]. However, reduced expression of MUC5AC in goblet cells from IPF lung lesions has been noted in comparison to controls [57,58]. Histone acetyltransferase p300, which is upregulated in IPF bronchial epithelial cells [97], reduces the expression of MUC5AC [98], as does the IPF-activated developmental Notch signalling pathway [99], which can directly downregulate MUC5AC promoter activity [100]. Furthermore, autophagy, which is defective in IPF [101,102,103], has been reported to increase the secretion of MUC5AC by promoting the phosphorylation of c-Jun N-terminal kinase (JNK) and c-Jun in patients with chronic rhinosinusitis [104]. By contrast, goblet cell metaplasia and hypersecretion of MUC5AC are common during respiratory tract inflammation, due to stimulation of fibrogenic cytokines such as interleukin (IL)-13 and EGF [96]. A putative NF-kB binding site in the promoter of MUC5AC has also been identified as critical to the induction of MUC5AC expression by IL-1β and IL-17A [105], which are both elevated in the bronchoalveolar lavage fluid (BALF) of IPF patients [106]. The cytokine IL-9, which is also involved in IPF disease [107], has been reported to activate MUC5AC via the the Janus kinase/signal transducers and activators of transcription (JAK/STAT) patway in respiratory epithelial cells [108] (Figure 4). Finally, neutrophil elastase, one key effector in the progression of lung fibrosis [109], increases MUC5AC mRNA levels by enhancing mRNA stability [110]. Nevertheless, there is no clear evidence about induction of MUC5AC expression during IPF acute exacerbations.

Recently, two tightly linked low-frequency MUC5AC single-nucleotide variants (SNVs) on the p-terminus of chromosome 11 (*rs34474233* and *rs34815853*) have been cited as risk factors for IPF. These variants result in the same missense amino acid change in MUC5AC, which was observed in 4.4% of controls and up to 13.8% of IPF patients. Thus, MUC5AC can be considered another biologically plausible IPF susceptibility gene [111]. However, further exploration of the influence of these SNVs on the expression, location and function of MUC5AC in IPF patients is needed.

### 3.3. Mucin 1

**Figure 5:**
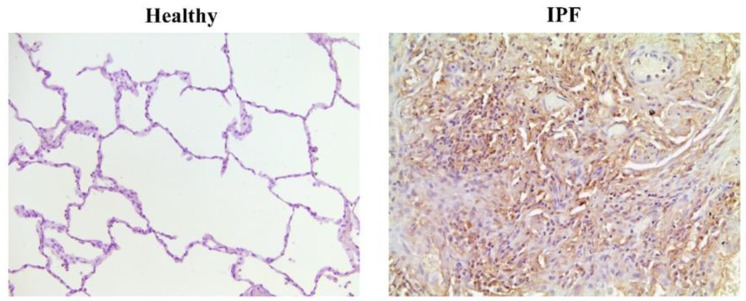
MUC1 expression in lung tissue from healthy and IPF subjects. Immunohistochemistry of MUC1 in lung tissue from healthy and IPF patients. In IPF patients, MUC1 expression is observed at hyperplastic alveolar type II cells and fibrotic areas. However, MUC1 is expression is almost undetectable in healthy subjects.

MUC1 is expressed at the basal level in most epithelial cells, including those around lung microvilli [112]. However, MUC1 has long been viewed as a tumour-associated molecule due to its frequent overexpression and aberrant glycosylation in many carcinomas (>90% of breast carcinomas, and frequently in other types of cancer, including ovarian, lung, colon, gastrointestinal and pancreatic carcinomas), where it is associated with metastatic and invasive potential [52,55,113]. The soluble form of MUC1 is also known as CA15-3 antigen, and is the most widely used diagnostic and prognostic serum marker in breast cancer [114,115]. In the context of IPF, overexpression of MUC1 has been observed in lung tissue from pulmonary fibrosis patients [59] (Figure 5). IPF itself increases the risk of lung carcinogenesis development [116], thus suggesting MUC1 as a feasible target for the treatment of this subgroup of IPF patients. Nevertheless, there is no direct evidence about the participation of MUC1 in patients presenting both pathologies.

**Figure 6:**
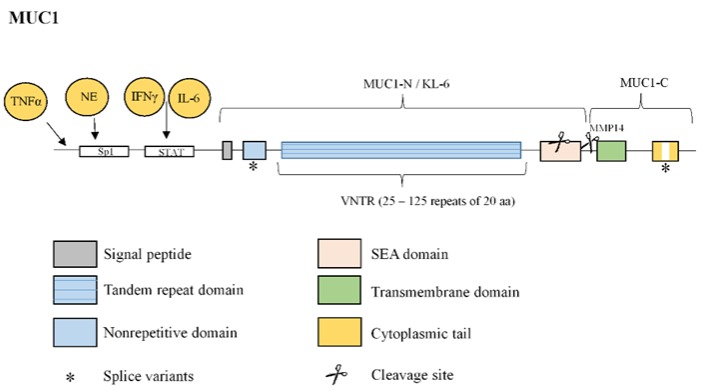
Structure of mucin MUC1. MUC1 protein is comprised of an N-terminal subunit (also called KL-6) constituted by an extracellular variable number tandem repeat (VNTR) domain composed of 25–125 repeats of 20 amino acids, and a C-terminal subunit constituted by a SEA domain, a transmembrane domain and a cytoplasmic tail (CT). MUC1 extracellular domain can be shed into the lumen by proteolytic cleavage in the SEA domain or by the action of metalloproteinase 14 (MMP14) in the region following the SEA domain. Two examples of splice variants of MUC1 transcript are shown (*): a nonrepetitive sequence insertion prior to the variable number tandem repeat (VNTR) region and variations in the CT. MUC1 overexpression is induced by activation of the single STAT-binding site of MUC1 promoter by interferon (IFN)γ or interleukin (IL)-6 or by an increased Sp1 binding to MUC1 promoter by neutrophil elastase (NE). Tumour necrosis factor α (TNFα) also upregulates the transcription of MUC1.

**Figure 7:**
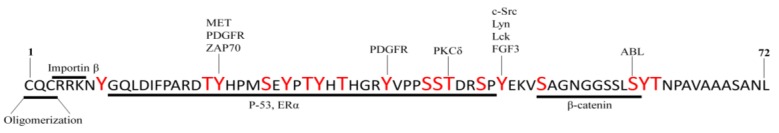
72 amino-acid sequence of the MUC1-C cytoplasmic domain. Amino acids in large, red font have been reported as phosphorylation sites and kinases are indicated. The CQC motif is necessary for MUC1-C oligomerization and the RRK motif is necessary for MUC1-C binding to importin β and targeting to the nucleus. Also highlighted is the β-catenin, p53 and estrogen receptor (ER) α binding sites.

MUC1 protein is comprised of a 20-amino acid residue extracellular TR, which is repeated in humans between 25 and 125 times, an SEA domain, a transmembrane domain, and a 72-amino acid C-terminal CT (Figure 6) that contains 18 documented potential and putative tyrosine or serine/threonine phosphorylation sites. This protein contributes to the modulation of multiple intracellular signals through interactions (largely regulated by CT phosphorylation) with various effectors implicated in proliferation, apoptosis, transformation and transcription of various genes [41,52,63,117,118] (Figure 7). At least 12 splice variants of the *MUC1* gene transcript have been described, and several structural modifications have been reported to result from these variations. For example, a 9-amino acid insertion prior to the VNTR region, which is predicted to alter the cleavage of its signal peptide, as well as variations in the intracellular region and loss of known signalling sites in the MUC1 intracellular region, have been observed [37] (Figure 6).

As a tethered mucin, MUC1 proteins can serve as a membrane receptor comprised of a MUC1 N-terminal subunit and a MUC1 C-terminal subunit, which form a stable non-covalent complex. Once at the plasma membrane, the MUC1 extracellular domain can be shed into the lumen through proteolytic cleavage of the SEA domain, which is a self-cleaving domain [119]. An alternative mechanism is proteolytic cleavage near the plasma membrane through the action of MT1-MMP or MMP14 [120,121] (Figure 6). Thus, the elevated levels of MT1-MMP under lung fibrotic conditions [122] are hypothesized to mediate the release of the extracellular MUC1 domain (MUC1-N/KL-6) in many IPF biological fluids. Several studies have reported increased serum KL-6 levels during acute exacerbation of IPF, and a recent study demonstrated that serial increases in serum KL-6 levels are correlated with a rapid decrease in predicted FVC, and also that higher KL-6 levels are correlated with lower IPF survival rates [11]. Therefore, secreted KL-6/MUC1 has been proposed as a useful biomarker for evaluating disease activity and predicting clinical outcomes of IPF [10]. Moreover, KL-6 has been confirmed to be a reliable prognostic biomarker indicative of the response to nintedanib treatment in IPF patients. Particularly, IPF patients treated with nintedanib for 12 months maintain stable FVC values and KL-6 levels [12]. KL-6 mechanism of action is not yet well understood. However, isolated KL-6 has been found to promote lung fibroblast migration, proliferation and transformation into myofibroblasts, as well as alveolar EMT [13,14]. Furthermore, preclinical studies have demonstrated that antibodies against KL-6 attenuate bleomycin-induced lung fibrosis [123], while MUC1 knockout (KO) mice exhibit resistance to bleomycin-induced pulmonary fibrosis, with improvements in lung function, survival and fibrotic lung tissue remodelling [64].

MUC1 overexpression is reportedly induced by interferon γ (IFN-γ) in ovarian and breast cancer cell lines [124]. This finding might explain, at least in part, the unsatisfactory results obtained in recent decades using IFN-γ for IPF therapy [125]. One possible explanation for the increase of MUC1 mRNA levels in breast carcinoma cells is activation of the single STAT-binding site of the MUC1 promoter by IFN-γ and fibrogenic IL-6 [126]. More recently, fibrogenic TNFα has been shown to upregulate the transcription of MUC1 in nasal epithelial cells [127]. Similar to MUC5AC, neutrophil elastase increases MUC1 expression, but only at the transcriptional level through increased binding of Sp1 to the MUC1 promoter [128] (Figure 6).

**Figure 8:**
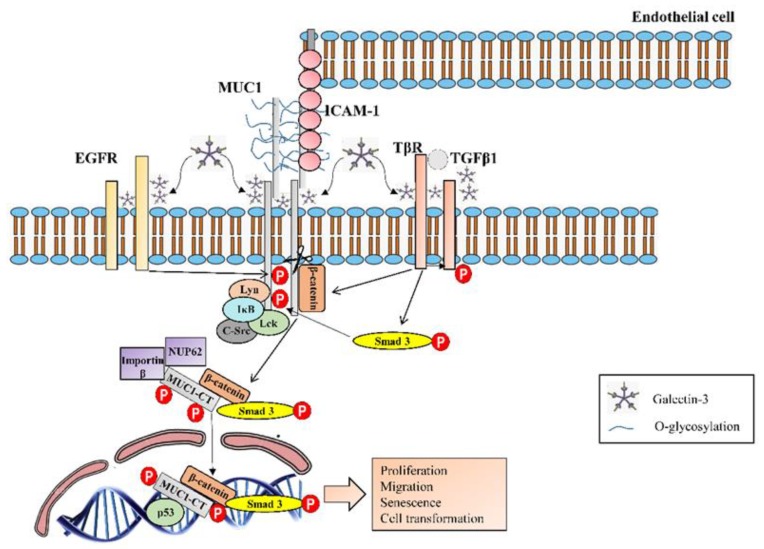
MUC1 activation and intracellular signalling. MUC1 is activated by extracellular ligands such as the intracellular adhesion molecule-1 (ICAM-1), which interacts with the protein component of the MUC1 VNTR or MUC1-N terminal domain. MUC1-C interacts with the galectin 3 ligand, which functions as a bridge to associate MUC1-C with different growth factor receptors at the cell surface as for example epidermal growth factor receptor (EGFR), which can activate and phosphorylate MUC1-CT. For the transforming growth factor β1 (TGFβ1) receptor (TβR), galectin-3 stabilizates it, so galectin-3 could serve also as a bridge between MUC1-C and TβR. Moreover, it has been hypothesized that through Smad3 phosphorylation, induced by TβR activation, MUC1-CT is phosphorylated and translocated into the nucleus. Furthermore, TGFβ1 is also able to activate and induce nuclear localization of the fibrotic transcription factor β-catenin. A nuclear protein complex constituted by MUC1-CT/phospho (p)-Smad3 and β–catenin is formed and it promotes fibrotic processes such as epithelial to mesenchymal transition (EMT) and fibroblast to mesenchymal transition (FMT). MUC1-CT can also directly interact with intracellular kinases such as c-Src, Lyn and Lck or IκB kinase complex, which also activate and phosphorylate MUC1-CT. Phosphorylated MUC1-CT interacts with importin-β and with nucleoporin p62 (Nup62) to migrate into the nucleus. Once in the nucleus, MUC1-CT forms also nuclear complexes with other proteins as for example p53 and mediates IPF-linked processes such as proliferation, migration, cell senescence and cell transformation.

MUC1 is activated by extracellular ligands such as intracellular adhesion molecule-1 (ICAM-1), which interacts with the protein component of the MUC1 VNTR or MUC1 N-terminal domain [60], and which was elevated in the serum of IPF patients [61]. This association results in a rapid increase of intracellular calcium in MUC1-expressing cells [129] and consequently cytoskeletal changes and motility. Additionally, MUC1-C interacts with the galectin 3 ligand, which functions as a bridge between MUC1-C and the EGF receptor (EGFR), and potentially to other cell-surface receptors [62] (Figure 8). Increased galectin-3 expression has been observed in the BALF and serum of patients with stable IPF compared to those with non-specific interstitial pneumonitis and controls [130]. Galectin-3 promotes IPF through stabilization of the transforming growth factor β1 (TGFβ1) receptor (TβR) [130], so galectin-3 could also serve as a bridge between MUC1-C and TβR. In this context, galectin-3 KO in vivo models and galectin-3 inhibitors block IPF progression [130]. These results support the function of MUC1 as a therapeutic target for IPF. Based on these findings, a phase I/II clinical trial (NCT02257177) to assess the safety, tolerability, pharmacokinetics and pharmacodynamics of TD139 (galectin 3 inhibitor) in IPF patients has been successfully conducted.

MUC1-CT is phosphorylated by several cell surface growth factor receptors involved in proliferation, apoptosis, transformation and transcription of various genes. For instance, platelet-derived growth factor receptor beta (PDGFRβ) [131], fibroblast growth factor receptor 3 (FGFR3) [132], and the Met [65] and ErbB1–4 receptors [63,133] are all able to phosphorylate MUC1-CT and thereby promote cell proliferation, migration and transformation [65,117] (Figure 7 and Figure 8). Moreover, it has been observed MUC1-CT collaboration with TGFβ1 (the most important IPF pro-fibrotic factor) to induce IPF progression. It has been hypothesized that MUC1-CT is phosphorylated and translocated into the nucleus through Smad3 phosphorylation, which is induced by TβR activation. Furthermore, TGFβ1 is able to activate and induce nuclear localization of the fibrotic transcription factor β-catenin. Then, a nuclear protein complex comprised of MUC1-CT/phospho (p)-Smad3 and active (act)-β-catenin is formed, which promotes fibrotic processes such as EMT and the fibroblast to mesenchymal transition (FMT) [64].

MUC1-CT can also directly interact with intracellular kinases such as c-Src, Lyn and Lck, which are activated by growth factors that are elevated in IPF [65], as well as with components of the IκB kinase (IKK) complex, triggering NF-κB overactivation and induction of anti-apoptotic functions and cellular transformation [65] (Figure 7 and Figure 8).

In terms of the detailed MUC1-CT phosphorylation mechanism, it has been observed that Thr41 (1224) phosphorylation at the TDR amino acid site by protein kinase Cδ (PKCδ), as well as Tyr46 (1229) phosphorylation at the YEKV amino acid site by c-Src, Lyn and Lck tyrosine kinases, FGF3, or EGFR [117], induce binding of the MUC1-CT SAGNGGSSLS sequence to β-catenin. This binding leads to nuclear translocation, promoting activation of Wnt target genes [134] and CTGF [135], which are both associated with IPF [136,137]. Moreover, the interaction of MUC1-CT/β-catenin is involved in in vivo mammary gland transformation and metastasis [65]. Similarly, Tyr phosphorylation by PDGFRβ induces nuclear localization of the MUC1-CT/β-catenin complex, determining the invasiveness of pancreatic adenocarcinoma cells and influencing WNT signalling and the transcription of cyclin D1, as well as the fibrotic process of EMT [35,138]. Other MUC1-CT phosphorylation inducers have been reported [117,139] and further investigation of its influence in cellular processes is needed (Figure 7).

MUC1-CT also includes a CQC sequence necessary for the formation of MUC1-C oligomers [63]. In carcinoma cells, MUC1-C accumulates in the cytoplasm as a result of release from the cell membrane, redirection from the endoplasmic reticulum or through a mechanism that depends on its oligomerisation [63,140]. Furthermore, MUC1-CT interacts with importin-β through the RRK motif, and with nucleoporin p62 for migration into the nucleus [65]. Once in the nucleus, MUC1-CT associates with certain transcription factors including β-catenin/TCF4, p53, estrogen receptor-α, NF-kB p65 and STATs [63], thereby promoting fibrotic processes (Figure 7).

As noted above, MUC1 is one of the best-studied tumour-associated antigens, and is abnormally expressed in several malignant conditions. MUC1-targeting vaccines are currently in various stages of clinical trials, including: TG4010 (its safety and activity have been evaluated in phase II studies in several types of solid tumours) [141], Tecemotide (currently undergoing phase III clinical trials for non-small cell lung cancer) [142] and PANVAC (currently undergoing phase II clinical trials against colon and breast cancer) [143]. Anti-MUC1 antibodies are also available against various cancers. For example, PankoMab is a humanized monoclonal antibody that recognizes the tumour-specific epitope of MUC1 and is currently undergoing phase II trials for ovarian cancer. Beyond global targeting of MUC1 or MUC1 tumour-associated antigen, targeting MUC1-CT and its nuclear translocation might represent a promising option for the treatment of several types of cancer, as well as IPF. Currently, a phase I/II trial (NCT02204085) using the MUC1-CT CQC motif inhibitor GO-203 in patients with relapsed or refractory acute myeloid leukaemia is underway. In the context of IPF, preliminary results using the structural analogue GO-201 have shown in vitro and in vivo attenuation of the development of pulmonary fibrosis (Milara, J. et al., unpublished data).

### 3.4. Mucin 4

MUC4 is localized mainly in the cilia [42]. In the same manner as MUC1, MUC4 overexpression in cancer promotes IPF-linked processes including myofibroblast transition, proliferation, migration and metastasis [52,55]. Thus, MUC4 overexpression may also eventually serve as a diagnostic and prognostic marker for numerous cancers, including pancreatic tumours [144], where its expression is associated with the metastatic phenotype [145], lung adenocarcinomas [146], and mass-forming intrahepatic cholangiocarcinoma, where its co-expression with ErbB2 is correlated with a short survival time [147]. Nevertheless, no studies have investigated MUC4 as a direct therapeutic target.

MUC4 shows greater allelic diversity than other mucin genes, with a very large uninterrupted TR region containing 48-bp repeat units [148]. Nearly two dozen distinct splice variants of the human *MUC4* gene have been identified, which encode an assortment of secreted and membrane-bound forms of the protein. For example, some splice events in the region downstream of the central TR domain have been identified based on deletions and insertions in this region. Alternative splicing lacking the TR domain or a high degree of polymorphism was observed in the central TR region, with allele sizes ranging from 23.5 to 10.0 kb [149,150]. Interestingly, the anti-adhesive function of MUC4, which eliminates cell–cell and cell–matrix interactions, is dependent on the number of these TRs [151].

**Figure 9:**
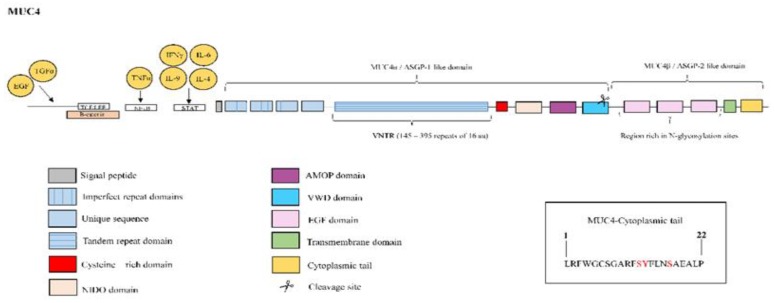
Structure of mucin MUC4. MUC4 protein is constituted by the ASGP-1-like extracellular domain or MUC4α, which contains an N-terminal signal sequence followed by: three imperfect repeats, an unique sequence, a variable number tandem repeat (VNTR) composed of 145–395 repeats of 16 amino acids, a cysteine-rich domain, nidogen-like domain (NIDO), adhesion-associated domain in MUC4 and other proteins (AMOP) and Von Willebrand factor type D domain (vWD). The cleavage site is located in this vWD domain. The ASGP-2-like or MUC4β subunit is rich in N-glycosylation sites and contains up to three EGF-like domains, a hydrophobic transmembrane region, and a 22-amino-acid cytoplasmic tail. The right box contains the amino acid sequence of the cytoplasmic tail with potential sites for phosphorylation marked in red color. MUC4 overexpression is induced by interleukin (IL)-9, IL-4 and IL-6 mediated JAK/STAT pathway activation. Combined treatment with tumour necrosis factor (TNF)α and interferon (IFN)-γ has been also shown to upregulate MUC4 expression through nuclear factor kappa B (NF-κB) and JAK/STAT pathways respectively. The growth factors epidermal growth factor (EGF) and transforming growth factor α (TGα) also upregulate MUC4 expression. Three T-cell factor/lymphoid enhancer factor (TCF/LEF)-binding sites on the MUC4 promoter and the binding of β-catenin has been demonstrated on MUC4 promoter.

MUC4 is derived from a single gene that is post-translationally processed into an O-glycosylated extracellular subunit of approximately 600–800 kDa, and a largely N-glycosylated fragment of approximately 120 kDa. This heterodimer is a homolog of ascites sialoglycoprotein (ASGP)-1 and ASGP-2, which form the rat sialomucin complex. The ASGP-1-like extracellular domain, or MUC4α, contains an N-terminal signal sequence followed by three imperfect repeats of 126–130 residues, a unique sequence, three putative functional domains including a TR domain of 16 amino acid residues repeated up to nearly 400 times, a cysteine-rich domain, nidogen-like domain (NIDO), adhesion-associated domain in MUC4 and other proteins (AMOP) and a von Willebrand factor type D (vWD) domain. The GDPH cleavage site is located in this vWD domain. Furthermore, the ASGP-2-like or MUC4β subunit is rich in N-glycosylation sites and contains up to three EGF-like domains, a hydrophobic transmembrane region, and a 22-amino-acid CT containing a single tyrosine residue and two serine residues susceptible to phosphorylation [41,42] (Figure 9).

Overexpression of MUC4 has been observed in the pulmonary arteries of patients with IPF and pulmonary hypertension (PH) [152]. This suggests that MUC4 inhibition could be a feasible option to attenuate pulmonary artery remodelling and consequently reduce the PH development associated with IPF, thus avoiding the unsatisfactory results obtained previously using vasodilation agents such as endothelin-1 receptor antagonists [153,154,155]. Recently, MUC4 overexpression was also observed in lung tissue from IPF patients, localized mainly to hyperplastic alveolar type II cells and fibrotic focal areas [66].

Multiple pathways that regulate MUC4 expression through the binding of various transcription factors or cytokines to the MUC4 promoter have been reported over the years [156,157]. For example, upregulation of MUC4 expression in respiratory epithelial cells through JAK/STAT pathway activation, mediated by IL-9 [158] and IL-4 [159], has been reported. Induction of MUC4 expression has also been reported, due to IL-6, in a STAT-dependent manner in gastric cancer cells [160]. All of these cytokines are involved in IPF disease [107,161,162]. Among growth factors, the MUC4 transcription is activated by EGF and TGF-α via the activation of EGFR [156], which is upregulated in IPF patients [163]. PKC-mediated activation of *MUC4* has been suggested as another pathway by which growth factors increase MUC4 expression in cancer cells [156]. Combined treatment with TNFα and IFN-γ has been shown to upregulate *MUC4* expression through the NF-κB and JAK/STAT pathways, respectively [156]. Recently, three T-cell factor/lymphoid enhancer factor (TCF/LEF)-binding sites have been identified on the MUC4 promoter, and binding of fibrotic transcription factor β-catenin/TCF4 to the MUC4 promoter has been observed in cells from pancreatic ductal adenocarcinoma, inducing its expression [164] (Figure 9). Furthermore, MUC4 induces nuclear translocation of β-catenin, promoting growth, metastasis and angiogenesis in pancreatic cancer [67]. Finally, similarly to MUC5AC, neutrophil elastase increases MUC4 levels through post-transcriptional mRNA stabilization [165].

**Figure 10:**
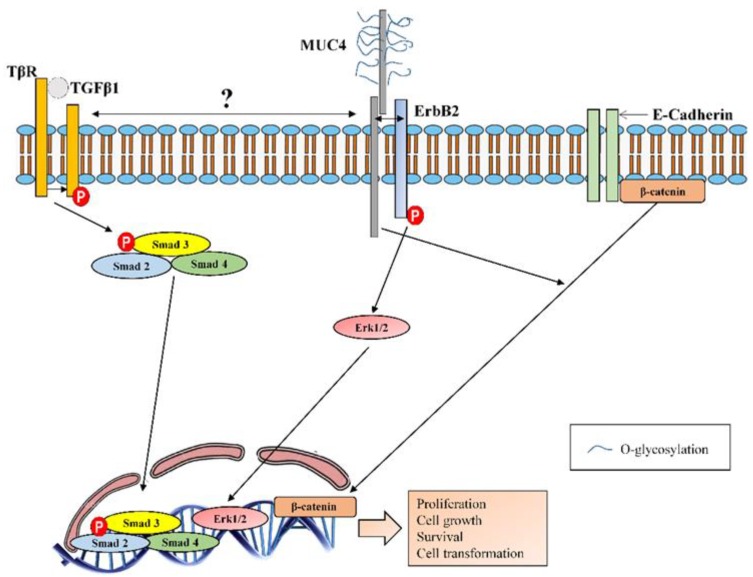
MUC4 molecular interactions. MUC4, via one of the EGF-like domains, serves as an intramembrane ligand for the receptor tyrosine kinase ErbB2. Binding of MUC4 to ErbB2 induces its phosphorylation and enhanced activation of extracellular signal-regulated kinases (Erks) 1 and 2, promoting proliferation, growth, survival and differentiation. MUC4 collaborates with transforming growth factor β1 (TGFβ1) to induce epithelial to mesenchymal transition (EMT) and fibroblast to mesenchymal transition (FMT) in IPF disease, although it is still unknown the exact mechanism of collaboration between MUC4 and TGFβ1 receptor (TβR). MUC4 also induces the nuclear translocation of β-catenin promoting growth, metastasis and angiogenesis.

MUC4 serves as an intramembrane ligand for ErbB2 receptor via one of its EGF-like domains [42]. Binding of MUC4 to ErbB2 may competitively inhibit the interaction of ErbB2 with its soluble ligands, in addition to inducing ErbB2 phosphorylation. In this manner, enhanced stabilization of ErbB2 through MUC4 interaction was associated with enhanced activation of the extracellular signal-regulated kinase (Erk) 1/2 MAPK pathway [68]. In this context, MUC4 could regulate cell proliferation, growth, survival and differentiation (as observed in breast cancer) in IPF. Moreover, MUC4 itself also modulates cell apoptosis, regulates cell–cell adhesion, and serves as a tumour-associated target for cancer therapy [37,42]. Experimental evidence has demonstrated that MUC4 overexpression in cancer cells is associated with increased expression of EMT-related transcription factors such as TWIST, ZEB1 and SNAIL [166]. Furthermore, MUC4 suppression in pancreatic cancer induces a change in the phenotype of cells, reducing the expression of mesenchymal cell-specific markers such as N-cadherin and vimentin and upregulating the expression of epithelial-specific markers such as cytokeratin-18, E-cadherin and occludin [167]. MUC4 has also been recently reported to collaborate with the fibrotic factor TGFβ1, inducing the EMT and FMT cellular transformations in IPF disease, although the exact mechanism of this collaboration remains unknown [66] (Figure 10).

### 3.5. Mucin 16

**Figure 11:**
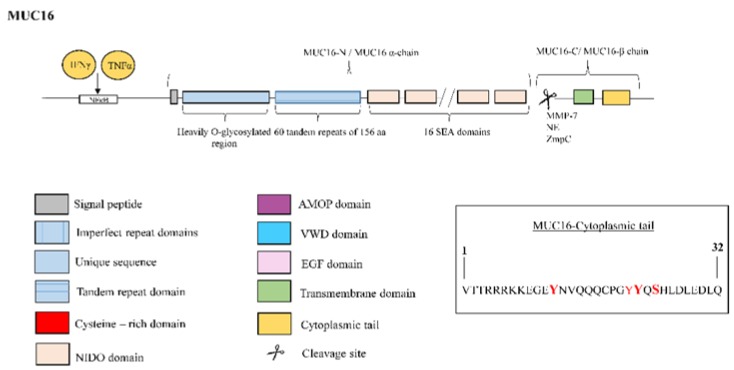
Structure of mucin MUC16. MUC16 protein is comprised by two subunits, the MUC16-N terminal or α subunit and the MUC16-C terminal or β subunit. The N-terminal subunit is constituted by a typical, heavily O-glycosylated mucin domain, 60 tandem repeats of 156 amino acids and 16 SEA modules. Otherwise, the C-terminal portion consists of a transmembrane (TM) region and a 32-residue cytoplasmic tail. The right box contains the amino acid sequence of the cytoplasmic tail with potential sites for phosphorylation marked in red color. For the amino acids in bold red font, phosphorylation was demonstrated. MUC16 clevage occurs in the 12 extracellular amino acids domain proximal to the TM domain. Proteases such as metalloproteinase-7 (MMP-7), neutrophil elastase (NE) and bacterial metalloprotease (ZmpC) have been implicated in enhanced shedding of MUC16 from the cell surface. Tumour necrosis factor (TNF)-α and interferon (IFN)-γ stimulate the expression of MUC16 through an nuclear factor kappa B (NF-κB) response element in the MUC16 promoter.

MUC16 is the largest of all known mucins. It is expressed in goblet cells, suggesting a role during initial contact as a first layer of defence [35]. Similarly to MUC1 and MUC4, MUC16 overexpression in cancer promotes EMT, cell proliferation, migration and metastasis [52]. In this context, fibrogenic TNF-α and IFN-γ stimulate the expression of MUC16 in breast, endometrial and ovarian cancers via an NF-κB response element in the MUC16 promoter [168] (Figure 11). However, the functional characteristics and signalling capabilities of MUC16 that contribute to these fibrotic processes in cancer remain unclear. Interestingly, the blood level of MUC16 in COPD patients was reported to be significantly higher than that of control subjects [169], while MUC16 mRNA transcript levels also show overexpression in lung tissue of IPF patients (unpublished results).

Yin and Lloyd [170] recently identified MUC16 as a TM mucin corresponding to the CA125 antigen, long known as a marker for ovarian cancer. This antigen is cleaved from the ovarian cancer cell surface into the bloodstream, promoting cancer cell proliferation and inhibiting the anti-cancer immune response [41]. Recently, CA125 was identified as a serum biomarker for disease progression and death in IPF patients [8]. However, the mechanism of CA125 generation remains to be elucidated.

As a tethered mucin, MUC16 is comprised of two subunits, the MUC16 N-terminal or α subunit and the MUC16 C-terminal or β subunit. The N-terminal subunit is a typical, heavily O-glycosylated mucin domain, containing 60 TRs of 156 amino acids and the SEA module. The C-terminal portion consists of a transmembrane region of 21 amino acid residues and a 32-residue CT with several tyrosine, serine, and threonine sites for potential phosphorylation [171]. In contrast to other tethered mucins, MUC16 contains 16 SEA modules, rather than just 1 [172,173]. Intriguingly, only the second MUC16 SEA domain resembles those found in MUC1 and other mucins. Cleavage of MUC16 does not occur at a conserved sequence in 1one of the SEA domains, and instead occurs at a domain of 12 extracellular amino acids proximal to the TM domain [174] (Figure 11). Consequently, a 17-kDa C-terminal fragment is released that contains the transmembrane domain, which is translocated to the nucleus and binds to chromatin, suggesting involvement in the regulation of gene expression [174]. Furthermore, proteases such as MMP-7, neutrophil elastase and bacterial metalloprotease (ZmpC) have been implicated in enhanced shedding of MUC16 from the cell surface [54,175].

**Figure 12:**
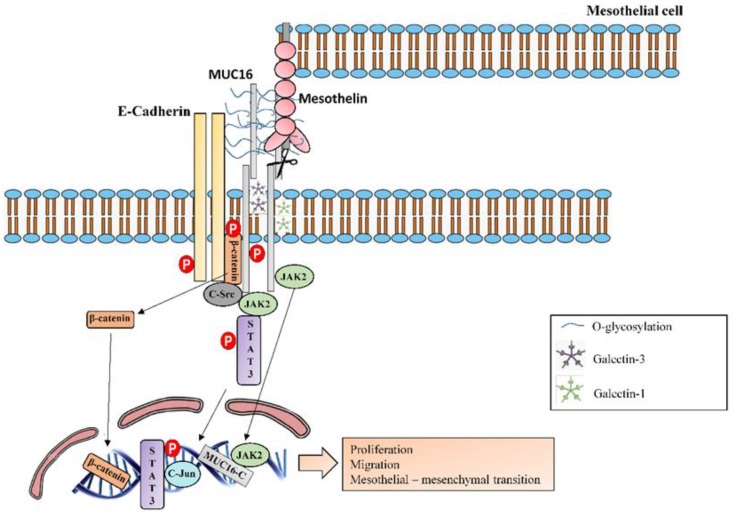
MUC16 molecular interactions and intracellular signalling. MUC16-C terminal subunit colocalizes into the nucleus with JAK2 and they both impart tumorigenic and metastatic functions. Furthermore, MUC16-C terminal subunit also associates with JAK2 in the cytoplasm and consequently activates STAT3 and c-Jun to promote cell proliferation. MUC16 has been observed associated with E-cadherin and β-catenin complexes and it inhibits GSK-3β-mediated phosphorylation and degradation of β-catenin, leading to increased levels of the fibrogenic transcription factor β-catenin. It is suggested that the cytoplasmic tail of MUC16 could bind to β-catenin and that the extracellular domain of MUC16 could interacts with the extracellular domain of E-cadherin and stabilizes E-cadherin at the cell membrane. MUC16-CT directly binds the c-src kinase, which phosphorylate it. C-src also mediates tyrosine phosphorylation of E-cadherin, inducing its ubiquitination and degradation. Otherwise, β-catenin phosphorylation by c-Src negatively regulates the binding to E-cadherin. Therefore, β-catenin is translocated into the nucleus. MUC16 extends from the surface and binds to mesothelin, a protein that is found on the surface of the mesothelial cells, activating MUC16 and enhancing epithelial mesothelial to mesenchymal transition. MUC16 has also been shown to bind with galectin-1 and galectin-3.

The MUC16 C-terminal subunit promotes nuclear translocation of JAK2, the expression of which is increased and activated in the lungs and pulmonary arteries of patients with IPF [176]; the nuclear co-localization of these substances imparts tumorigenic and metastatic processes in pancreatic cancer cells [177]. Furthermore, the association of JAK2 with the MUC16 C-terminal subunit has been shown to activate STAT3 and c-Jun, as well as to promote cell proliferation in breast cancer cells [69] (Figure 12). The C-terminal subunit is also able to decrease apoptosis through the TRAIL (tumour necrosis factor-related apoptosis-inducing ligand)-mediated extrinsic apoptotic pathway by reducing expression of the TRAIL receptor R2 [70]. Lung fibroblasts avoid apoptosis in IPF [178]. However, upregulation of TRAIL and its cognate receptors in alveolar epithelial cells (AECs) within the fibrotic lesions of IPF-affected lungs has been reported, indicating that TRAIL-mediated AEC apoptosis is a key event in IPF pathogenesis [179].

MUC16 has also been observed to associate with the E-cadherin and β-catenin complexes [180], and it inhibits GSK-3β-mediated phosphorylation and degradation of β-catenin. This process leads to increased levels of the fibrogenic transcription factor β-catenin, which promotes the formation of multicellular aggregates [181]. It has been suggested that the CT of MUC16 could bind to β-catenin despite lacking the β-catenin binding motif [181]. Moreover, it has been observed that MUC16 alters E-cadherin cellular localization and expression, and it has been suggested that the extracellular domain of MUC16 could interact with the extracellular domain of E-cadherin, stabilizing E-cadherin in the cell membrane [181]. MUC16-CT has been reported to directly bind to c-src kinase, which phosphorylates Tyr-22142 [71] (Figure 12). However, it remains unclear whether phosphorylation of this tyrosine plays a role in intracellular signalling events [71]. Src also mediates tyrosine phosphorylation of E-cadherin, inducing its ubiquitination and degradation [182]. Furthermore, β-catenin phosphorylation by c-Src negatively regulates its binding to E-cadherin [183]. These results suggest that MUC16 recruits more c-Src into E-cadherin-mediated cell–cell adhesion sites through interactions with c-Src and the E-cadherin/β-catenin complex, leading to deregulation of E-cadherin, enhanced cell migration and invasion of epithelial cancer cells [71].

As a large, heavily glycosylated molecule, MUC16 extends from the surface of ovarian cancer cells and binds to mesothelin, a protein that is found on the surface of mesothelial cells lining the peritoneum [52]. The MUC16-mesothelin interaction is capable of activating MUC16, thus enhancing ovarian cancer cell metastasis and the epithelial mesothelial to mesenchymal transition, suggesting that the binding of these proteins triggers pathways that regulate cellular adhesion and motility [42]. Binding of mesothelin to MUC16 markedly enhances pancreatic cancer cell motility and invasion through selective induction of MMP-7 (one of the molecules most highly expressed in IPF compared to normal lung tissues or those from other ILDs [122]) via a p38 MAPK-dependent pathway [184]. Recently, pleural mesothelial cells have been proposed as the potential origin of IPF myofibroblasts [185,186,187]. Thus, MUC16 activation in the pleural mesothelium may trigger IPF progression through the MUC16-mesothelin association, supporting this interaction as a potential therapeutic target for IPF. MUC16 has also been shown to bind with galectin-1, a mammalian lectin expressed by human immune cells, preventing anti-tumour immune responses [72]. Galectin-1 transcript levels increase in the lungs of IPF patients [188], and although the biological significance of the galectin-1/MUC16 interaction is not fully understood, galectin-1 has been reported to promote the development of hypoxia-induced pulmonary fibrosis [188]. Moreover, like MUC1, MUC16 interacts with galectin-3 in a galactose-dependent manner [73] (Figure 12).

As noted above, MUC16/CA125 is critically important in ovarian cancer. Therefore, antibodies against CA125, such as oregovomab and abagovomab, have been used in clinical trials with ovarian cancer patients, although without any positive outcomes [189,190]. These antibodies bind to the extracellular portion of MUC16 (i.e., CA125), such that it is highly likely that they never reach cancer cells. Therefore, preventing cleavage of MUC16 should be considered an alternative to existing MUC16-based therapeutic approaches, as it would increase the presence of MUC16 on the cell surface and reduce the nuclear functions of MUC16-CT [191].

## 4. Conclusions

As a consequence of the complexity of IPF, diagnosis and treatment thereof remains challenging. Nevertheless, continuous advances in our understanding of the pathophysiological process of this disease have led to the development of numerous anti-fibrotic therapies. However, only two treatments (pirfenidone and nintedanib) are currently approved, and both have limited efficacy. As lung mucins are broadly associated with the airway defence, cell growth and tissue remodelling processes compatible with the processes observed in IPF disease, it is reasonable to analyse the expression, distribution, activation and function of mucins in pulmonary fibrosis, which might provide a useful tool for IPF diagnosis, follow-up and treatment. However, mucin biology is complex; thus, in-depth study is required before mucins can be considered as a new therapeutic approach for IPF.

## Figures and Tables

**Table 1:** Summary of main potential serum biomarkers in idiopathic pulmonary fibrosis (IPF).

Biomarker	Definition	Significance
**Alveolar Epithelial Cell Dysfunction**		
KL-6 ^1^/MUC1	Glycoprotein mainly expressed at the extracellular membrane surface of type II pneumocytes [10].	Increased serum levels during IPF acute exacerbation [11]. Serum levels correlate with IPF severity and prognosis [11]. Biomarker indicative of the response to nintedanib treatment [12]. Promotion of lung fibroblast migration and proliferation, FMT ^2^ and EMT ^3^ [13,14].
CA15-3	Central protein core of MUC1	Serum levels significantly higher in patients with IPF [9]. Elevated serum levels correlate with decreased total lung capacity, decreased diffusing capacity of carbon monoxide and high resolution computed tomography findings [9].
CA125	Peptide epitope of MUC16	Rising concentrations over 3 months are associated with increased risk of IPF mortality [8].
Surfactant proteins (SP-A, SP-D and SP-C)	Lipoprotein complexes synthesized and secreted by type II pneumocytes.	Elevated serum levels in IPF patients [15]. Serum SP-A and SP-D levels are predictors of IPF prognosis [16,17]. Mutations on the genes encoding for SP-C and SP-A2 have been described within families of patients with pulmonary fibrosis [18].
MUC5B	Secreted mucin produced mainly in mucous cells of the submucosal glands [19].	A common gain-of-function promoter variant (*rs35705950*) has been reported in 30–35% of IPF patients [20].
**Extracellular Matrix Remodeling and Fibroproliferation**		
Matrix metalloproteinases (MMP-1 and MMP-7)	Zinc-dependent peptidases that are mainly responsible for ECM ^4^ degradation.	Elevated levels in the plasma, BALF ^5^ and tissue of IPF patients [21]. Elevated MMP-7 serum levels correlate with disease severity [21]
LOXL2 ^6^	Enzymes that facilitate the cross-linking of type 1 collagen molecules and stabilizes ECM.	Serum levels are correlated to IPF progression [22].
Periostin	Protein secreted by bronchial epithelial cells and that promotes ECM deposition and mesenchymal cell proliferation.	Elevated serum levels in IPF patients. Serum levels correlate with IPF physiological progression [23]
**Immune Disfunction**		
CCL18 ^7^	Small protein mainly secreted by monocytes, macrophages and dendritic cells that acts as a chemoattractant [24] and has an important role stimulating fibroblasts to synthesise collagen in fibrotic lung diseases [25].	Serum level is a predictor of IPF outcome and mortality [26].
IL-8 ^8^	Cytokine highly chemo-attractant for neutrophils	Negative correlation between IL-8, pulmonary function tests [27] and survival [28].
YKL-40	Chitinase-like protein produced from alveolar macrophages and type II pneumocytes which regulate proliferation of different cell types.	Serum and BALF YKL-40 levels are predictors of IPF survival [29]
TLR3 ^9^	Receptors that mediate the innate immune response to infection and tissue injury [30].	TLR3 L412F polymorphism is associated with a significantly greater risk of mortality and an accelerated decline in FVC ^10^ [31].
TLR9 ^11^	Receptors that mediate the innate immune response to infection and tissue injury [30].	Higher concentrations of TLR9 in surgical lung biopsies from IPF rapidly progressive patients than in tissue from IPF slowly progressing patients [32].
TOLLIP ^12^	Inhibitory adaptor protein within TLRs involved in the regulation of the innate immune system.	Significant correlation between response to N-acetylcysteine therapy and the *rs3750920* polymorphism [33]. The *rs5743890* minor allele is protective and associated with reduced susceptibility to IPF [34].

^1^ KL-6: Krebs von den Lungen-6; ^2^ FMT: fibroblast to mesenchymal transition; ^3^ EMT: epithelial to mesenchymal transition; ^4^ ECM: extracellular matrix; ^5^ BALF: bronchoalveolar lavage fluid; ^6^ LOXL2: lysyl oxidase-like 2; ^7^ CCL18: CC chemokine ligand 18; ^8^ IL-8: interleukin-8; ^9^ TLR3: Toll-like receptor 3; ^10^ FVC: forced vital capacity; ^11^ TLR9: Toll-like receptor 9; ^12^ TOLLIP: Toll-interacting protein.

**Table 2:** Classification, chromosome localization and main tissue expression of human mucins [39,40].

Mucin	Chromosome	Main Tissue Expression
**Secreted Mucins-Gel-Forming**		
MUC2	11p15.5	Jejunum, ileum, colon, endometrium, respiratory tact
MUC5AC	11p15.5	Respiratory tract, stomach, conjunctiva, endocervix, endometrium
MUC5B	11p15.5	Respiratory tract, submandibular glands, endocervix
MUC6	11p15.5	Stomach, ileum, gall bladder, endocervix, endometrium
MUC19	12q12	Sublingual gland, submandibular gland, respiratory tract, eye, middle ear epithelium
**Secreted Mucins-Non-Gel-Forming**		
MUC7	4q13-q21	Respiratory tract, sublingual and submandibular glands
MUC8	12q24.3	Respiratory tract, uterus, endocervix, endometrium
MUC9	1p13	Fallopian tubes
**Transmembrane Mucins**		
MUC1	1q21	Breast, pancreas, duodenum, ileum, colon, trachea, bronchi, cornea, conjunctiva, fallopian tubes, uterus, endometrium, endocervix, ectocervix, vagina
MUC3A/B	7q22	Small intestine, colon, gall bladder
MUC4	3q29	Breast, respiratory tract, small intestine, colon, conjunctiva, cornea, endocervix, ectocervix, vagina, endometrium, lungs
MUC12	7q22	Colon, small intestine, stomach, pancreas, lung, kidney, prostate, uterus
MUC13	3q21.2	Colon, trachea, kidney, small intestine
MUC14	4q24	Heart, kidney, lungs
MUC15	11p14.3	Colon, respiratory tract, small intestine, prostate
MUC16	19p13.2	Ovary, cornea, conjunctiva, respiratory tract, endometrium
MUC17	7q22	Stomach, duodenum, colon
MUC20	3q29	Placenta, colon, respiratory tract, prostate, liver
MUC21	6p21	Respiratory tract, thymus, colon
MUC22	6p21.22	Lungs, placenta, testis

**Table 3:** Summarizes the main roles of the major lung mucins in IPF.

Mucin	Participation in IPF
**Secreted Mucins-Gel-Forming**	
MUC5B	Expression 14.1 higher in IPF patients than in control subjects [20]. *rs35705950*: risk allele for IPF development (30%–35% of IPF patients) [20]. *rs35705950*: survival advantage allele [56].
MUC5AC	Reduced expression in globet cells from IPF lesions in comparison with controls [57,58]. *rs34474233* and *rs34815853*: risk alleles for IPF development.
**Transmembrane Mucins**	
MUC1	Overexpression in lung tissue from IPF patients [59]. Secreted KL-6 ^1^/MUC1 is proposed as a useful biomarker to evaluate disease activity and predict the clinical outcomes in IPF [10]. Secreted MUC1/KL-6 promotes lung fibroblast migration, proliferation, EMT ^2^ and FMT ^3^ [13,14]. MUC1 is activated by the extracellular endothelial ICAM-1 ^4^ [60], elevated in serum of IPF patients [61]. MUC1-C terminal subunit interacts with the fibrotic galectin-3, serving as a bridge to associate MUC1-C with cell surface growth receptors involved in IPF [62]. Cell surface growth factor receptors involved in IPF (such as EGFR ^5^, FGFR3 ^6^, PDGFR ^7^ and TGβR ^8^) phosphorylate and activate MUC1-CT ^9^ [63,64]. MUC1-CT is phosphorylated and activated by intracellular kinases such as c-Src, Lyn and Lck, which are activated by growth factors elevated in IPF [65] MUC1-CT interacts with the fibrotic transcription factor β-catenin and they both together translocate into the nucleus [64] MUC1-CT nuclear translocation promotes fibrotic processes [63].
MUC4	MUC4 is overexpressed in lung tissue from IPF patients [66]. MUC4 collaborates with the fibrotic TGFβ1 to induce EMT and FMT cellular transformations [66]. MUC4 induces nuclear translocation of the fibrotic transcription factor β-catenin [67]. MUC4 serves as an intramembrane ligand for ErbB2, inducing activation of ERK ½ ^10^ and regulating cell proliferation, growth, survival and differentiation [68].
MUC16	MUC16/CA125 antigen has been identified as a serum biomarker to predict disease progression and death in IPF patients [8]. MUC16-C terminal subunit associates with JAK2 ^11^ and promotes cell proliferation [69]. MUC16-C terminal subunit decreases TRAIL ^12^-induced apoptosis [70]. MUC16 interacts with c-Src and the E-cadherin/β-catenin complex, leading to deregulation of E-Cadherin and enhancing cell migration [71]. MUC16 binds to mesothelin, enhancing epithelial mesothelial to mesenchymal transition, cell motility and invasion [42]. MUC16 binds to galectin-1 [72] and galectin-3 [73].

^1^ KL-6: Krebs von den Lungen-6; ^2^ EMT: epithelial to mesenchymal transition; ^3^ FMT: fibroblast to mesenchymal transition; ^4^ ICAM-1: intercellular adhesion molecule 1; ^5^ EGFR: epidermal growth factor receptor; ^6^ FGFR3: fibroblast growth factor receptor 3; ^7^ PDGFR: platelet derived growth factor receptor; ^8^ TGβR: transforming growth factor β receptor; ^9^ MUC1-CT: MUC1-cytoplasmic tail; ^10^ ERK1/2: extracellular signal-regulated kinase 1/2; ^11^ JAK2: Janus kinase 2; ^12^ TRAIL: TNF-related apoptosis-inducing ligand.
